# Use of Bacterial DNA Concentration in Ascites as a Marker for Spontaneous Bacterial Peritonitis

**DOI:** 10.1016/j.jceh.2024.101434

**Published:** 2024-05-01

**Authors:** Niklas F. Aehling, Arno Hagenunger, Sandra Krohn, Katharina Zeller, Kathrin Jäger, Adam Herber, Cornelius Engelmann, Thomas Berg

**Affiliations:** ∗ Division of Hepatology, Department of Medicine II, Leipzig University Medical Center, Germany; † Department of Gastroenterology, Universitaetsklinikum Augsburg, Augsburg, Bayern, Germany; ‡ IZKF-FACS-Core Unit, Leipzig University, Leipzig, Germany; § Liver Failure Group, Institute for Liver and Digestive Health, University College London, Royal Free Campus, London, United Kingdom; ‖ Department of Hepatology and Gastroenterology, Campus Virchow-Klinikum, Charité - Universitaetsmedizin Berlin, Berlin, Germany; ¶ Berlin Institute of Health (BIH), Berlin, Germany

**Keywords:** ascites, bacterial DNA, spontaneous bacterial peritonitis, liver cirrhosis, IL-6

## Abstract

**Background and aims:**

Spontaneous bacterial peritonitis (SBP) is a common and serious complication in patients with decompensated cirrhosis. Precise quantification of bacterial DNA (bactDNA) and the related inflammatory response might add further information on the course of disease. The aim of the study was to evaluate the association between bactDNA, cytokine levels and clinical outcome.

**Methods:**

Ascites and serum samples of 98 patients with decompensated liver cirrhosis (42 with SBP and 56 without SBP) as well as serum samples of 21 healthy controls were collected. BactDNA in ascites and serum was detected and quantified by 16S rRNA PCR. Concentrations of IL-1β, TNF-α, IL-6, IL-8 and IL-10 were measured by a LEGENDplexTM multi-analyte flow assay. Clinical data were collected and analyzed retrospectively.

**Results:**

BactDNA was detected more frequently in ascites of patients with SBP (*n* = 24/42; 57.1%) than in ascites of patients without SBP (*n* = 5/56; 8.9%; *P* < 0.001). Additionally, IL-6 levels in both ascites and serum were significantly higher in patients with SBP (ascites *P* < 0.001, serum *P* = 0.036). The quantity of bactDNA in ascites was strongly correlated with polymorphonuclear neutrophil count in ascites (r = 0.755; *P* < 0.001) as well as ascites IL-6 levels (r = 0.399; *P* < 0.001). Receiver operating characteristic (ROC) curve analysis to diagnose SBP provided an AUC of 0.764 (95% CI: 0.661–0.867) for serum IL-6 levels, an AUC of 0.810 (95% CI: 0.714–0.905) for ascites IL-6 levels, and an AUC of 0.755 (95% CI: 0.651–0.858) for bactDNA levels in ascites.

**Conclusions:**

The correlation between the amount of bactDNA and IL-6 confirms the pathophysiological relevance of bactDNA and IL-6 as potential biomarkers for the diagnosis of SBP.

Spontaneous bacterial peritonitis (SBP) is a common and severe complication accounting for ∼10–30% of infections in hospitalized patients with decompensated liver cirrhosis and ascites.^1^^,^^2^ Although in-hospital mortality associated with SBP has decreased in the past decades from 38% to 17.6%,^3^ SBP remains a significant risk factor for fatal disease courses.^4^

Current guidelines for diagnosing SBP recommend performing paracentesis with subsequent manual microscopic or automated count of polymorphonuclear neutrophils (PMN) in ascites. A PMN cut-off of 250/μL has the greatest sensitivity for detecting of SBP, whereas a PMN cut-off of 500/μL has the greatest specificity.^5^ Ascites microbial culture is usually performed to detect the causative pathogens, but its sensitivity remains low with more than 50% negative results. In some cases (∼3–10%), ascites culture provides positive results despite low PMN count (<250/μL)^6^^,^^7^ which is defined as bacterascites.^8^ Data about the clinical significance of bacterascites are conflicting,^9^, ^10^, ^11^ but a recent study^12^ confirmed that bacterascites is associated with high short-term mortality of 32% in 30 days, if not treated adequately, most probably due to the risk of progressing to overt SBP. Early and adequate pathogen-specific therapy after initial broad-spectrum antibiotic treatment is crucial to lower the mortality of patients with SBP.^13^ Therefore, detection of bacterial pathogens in ascites is highly relevant, but there is still a lack of reliable diagnostic tools in clinical practice.^14^^,^^15^ An alternative to the conventional ascites culture is the detection and characterization of bacterial DNA (bactDNA) based on 16S rRNA genes. Results from previous studies were often controversial and frequently yielded false positives^16^, ^17^, ^18^, ^19^ most likely due to contaminations. We recently established a method based on real-time PCR and subsequent sequencing with ultra-clean reagents, which avoided contaminations and thus provided a high sensitivity and specificity to detect pathogens in ascites.^20^ A strong association between the level of bactDNA in ascites and the occurrence of SBP as well as the 30-day and 180-day survival could be demonstrated.^2^ Therefore, it is likely that there is a link between bactDNA and local and systemic inflammatory response.

The objective of the current study was twofold. Firstly, we aimed to investigate the potential correlation between inflammatory markers and the presence of bacterial DNA (bactDNA) in patients with cirrhosis, both with and without spontaneous bacterial peritonitis (SBP). Secondly, we aimed to assess the impact of bactDNA on the prognosis and clinical progression of patients with decompensated liver cirrhosis.

## METHODS

### Study Design

We performed a retrospective analysis of ascites and serum samples from 98 patients with decompensated cirrhosis from our biobank at the University Hospital Leipzig, which were collected simultaneously between March 2011 and July 2019. Forty two patients had SBP with an ascites PMN count >250/μL. In addition, 21 healthy individuals without liver disease or chronic inflammatory diseases were included as a control group and matched 1:2 with the SBP group by age and gender. Because this study was exploratory, no sample size calculation was performed. Patients under the age of 18 and patients with antibiotic therapy for SBP at the time of paracentesis were excluded. Patients with antibiotic therapy for non-SBP infections and with prophylactic antibiotic treatment were included. Furthermore, patients with previous episodes of SBP were not included in the non-SBP group. In case SBP was diagnosed, antibiotic therapy was initiated according to international guidelines.^21^ All patients gave written informed consent. The study protocol conformed to the ethical guidelines of the 1975 Declaration of Helsinki and was approved by the local ethics committee (No.: 356_10-ek_290817).

Clinical and laboratory parameters were assessed at baseline. Patients were followed for 360 days in order to assess the rate of liver transplantations together with the development of cirrhosis-associated complications such as gastrointestinal bleeding, hepatic encephalopathy, the need for subsequent paracentesis, infections other than SBP, ACLF and death.

### Sampling of Ascites and Serum

50 mL ascites was collected from each patient under sterile conditions and subsequently centrifuged at 4029 g for 25 min at room temperature. The ascites pellet was resuspended in 3 mL ascites supernatant with 20% glycerol. Ascites fluid supernatant and ascites pellet were stored at −20 °C until further analysis. Whole blood and serum samples were taken within one day of paracentesis and stored at −20 °C with 20% glycerol until further analysis. Ascites fluid supernatant and serum were used for the quantification of cytokines. Ascites pellet and whole blood served for the quantification of bactDNA.

### Quantification of Cytokines

Cytokines were analyzed in 25 μL serum and 25 μL ascites supernatant samples by a customized LEGENDplex™ (BioLegend, Koblenz, Germany) multi-analyte flow assay to quantify IL-1β, TNF-α, IL-6, IL-8 and IL-10 according to manufacturer's instructions. Detection beads were sonicated before serum or ascites supernatant samples were added. Beads bound to target cytokines were washed, incubated with detection antibodies and then washed again. Fluorescence intensity was quantified using a BD LSR II (Becton Dickinson and Company Biosciences, Franklin Lakes, USA) flow cytometer with application of a standard curve. Results were analyzed using the LEGENDplex software v8 (BioLegend). Test samples were analyzed in duplicates and diluted if necessary.

### Analysis of Bacterial DNA

BactDNA was isolated from the resuspended ascites cell pellet and whole blood using the ultra-clean MolYsis Complete5 (Molzym, Bremen, Germany) DNA isolation kit in two ways: 1. BactDNA from intact bacterial cells was isolated according to manufacturer's instructions including a DNase pre-treatment to diminish the human DNA background, using 1 mL of the resuspended ascites cell pellet sample for isolation of bactDNA from ascites or 1 mL of the whole blood sample for isolation of bactDNA from blood. 2. Quantification of bactDNA was generated by an ascites isolation series from 1 mL of the resuspended ascites cell pellet sample and a blood isolation series from 1 mL of the whole blood sample without DNase pre-treatment. In our study, this second series was used for the subsequent PCR because it was more reliable. Each isolation series included a negative control (fetal calf serum) and a positive control (*E. coli* spike-in).

Detection and differentiation of the bacterial genera and subsequent quantification of bactDNA was performed as previously described^20^ using ultra-clean Molzym Complete PCR Mastermix (Molzym, Cat. No. S-020-0250). The V3/V4 variable region of the 16S rRNA gene was covered with broad range bacterial primers and real-time PCR detection based on Sybr green was performed in duplicates with negative and positive PCR controls. With this method and by using the appropriate primers, it was also possible to detect infections caused by mixed pathogens. All analyzed samples with adequate melting-curve temperature and amplicon size after gel electrophoresis were considered positive. All DNA isolation steps and PCR analyses were performed in a HEPA-filtered hood with daily UV-radiation. The primer sequences are listed in Supplementary Table 1.

### Statistical Analysis

Statistical analysis was performed using SPSS 24 (SPSS, Illinois, USA). Categorical variables were displayed as frequencies (%), continuous variables as mean ± standard deviation or median (range), as appropriate. Chi-square test was applied for nominal variables and Mann-Whitney-U test was applied for metric variables. *P*-values below 0.05 were considered statistically significant. Kaplan–Meier curves were used for survival analysis, followed by performing the Wilcoxon test to compare survival curves. Correlations were calculated using the Pearson correlation coefficient. Only significant correlations with *r* > 0.3 were considered. Additionally, the accuracy of respective markers in the diagnosis of SBP was assessed by calculating the area under the receiver operating characteristic curve (AUROC). We used Cox regression analysis adjusting for age, gender and MELD score to assess factors modifying the cause-specific hazard functions censoring time to event endpoints. A STARD 2015 checklist is attached as Supplementary Table 5.

## RESULTS

### Patient Characteristics

In total, 42 out of 98 patients (42.9%) were diagnosed with SBP. There was no significant difference between patients with and without SBP in terms of age and gender. Epidemiological data are summarized in Table 1.

**Table 1:** Epidemiological Data.

	SBP (*n* = 42)	Non-SBP (*n* = 56)	*P*-value^a^	Controls (*n* = 21)	*P*-value^b^
Age (y)	56.5 (33–79)	60 (28–79)	0.306	54 (45–71)	0.483
Gender	♂ 29 (69.0) ♀ 13 (31.0)	♂ 41 (73.2) ♀ 15 (26.8)	0.659	♂ 12 (57.1) ♀ 9 (42.9)	0.206
BMI (kg/m^2^)	25.4 (14–42.9)	26.7 (16.7–40.9)	0.570	n.s.	

SBP: Spontaneous bacterial peritonitis; BMI: Body mass index.

a SBP group vs. non-SBP group.

b Controls vs. all cirrhosis patients; n.s., not stated.

The main cause of liver cirrhosis was alcohol-related liver disease (ALD) (*n* = 72, 73.5%). Patients with and without SBP did not differ significantly in severity of cirrhosis according to Child-Pugh-classification (*P* = 0.322), model of endstage liver disease-score (MELD) (*P* = 0.117) or the occurrence of hepatocellular carcinoma (HCC) (*P* = 0.757). Patients with SBP had a higher average CLIF-C-ACLF Score (57 vs 44; *P* < 0.001) than non-SBP patients (Table 2). Differences in clinical and laboratory data, including serum inflammation markers, are shown in Table 2.

**Table 2:** Baseline Characteristics.

Parameter; Median (range) or *N* (%)	SBP group (*n* = 42)	Non-SBP group (*n* = 56)	*P*-value
Etiology of cirrhosis			0.430
*ALD*	30 (71.4)	42 (75.0)	
*Viral (hepatitis B and C)*	2 (4.8)	2 (3.6)	
*MASH*	0 (0.0)	3 (5.3)	
*Other*	10 (23.8)	9 (16.1)	
Child–Pugh classification			0.332
*Class A*	3 (7.9)	1 (1.8)	
*Class B*	14 (36.8)	25 (44.6)	
*Class C*	21 (55.3)	27 (48.2)	
MELD score	19.5 (6–40)	18 (8–38)	0.117
*6–11 points*	4 (9.5)	6 (10.7)	
*12–24 points*	22 (52.4)	39 (69.7)	
*25–40 points*	16 (38.1)	11 (19.6)	
Hepatocellular carcinoma	6 (14.3)	6 (10.7)	0.757
EF CLIF ACLF grade			**0.035**
*No ACLF*	21 (50.0)	40 (71.4)	
*Grade 1*	13 (31.0)	14 (25.0)	
*Grade 2*	7 (16.6)	2 (3.6)	
*Grade 3*	1 (2.4)	0 (0)	
EF CLIF OF score	7.5 (6–12)	7.0 (6–11)	**0.011**
CLIF-C-ACLF score	57 (40–71)	44 (34–56)	**<0.001**
Use of beta-blockers	15 (35.7)	27 (48.2)	0.302
Use of antibiotics			
*Prophylactic*	9 (21.4)	6 (10.7)	0.166
*At time of paracentesis*	11 (26.2)	0 (0.0)	**<0.001**
Use of PPIs	28 (66.7)	39 (69.6)	0.828
Use of immunosuppressants	2 (4.8)	2 (3.6)	1.000
Mean arterial pressure (mmHg)	79.3 (53.7–120.0)	85.2 (56.7–112.3)	0.914
Heart rate (beats/minute)	79.3 (54–120)	80.0 (37–120)	0.521
Temperature (°C)	36.5 (36–39)	36.4 (35.5–38.2)	0.546
Bilirubin (μmol/L)	42.3 (6.2–593.8)	44.4 (11.7–330.3)	0.067
Creatinine (μmol/L)	131.0 (63–543)	115.5 (43–411)	**0.026**
GFR (mL/min; CKD-EPI formula)	43 (8–105)	56 (13–135)	**0.029**
INR	1.4 (0.9–4.9)	1.5 (1.0–5.2)	0.822
Albumin (g/L)	30.4 (13.8–48.0)	28.3 (13.9–44.5)	**0.035**
WBC count (exp9/L)	9.7 (2.7–33.2)	6.0 (1.4–17.1)	**<0.001**
CRP (mg/dL)	67.0 (5.42–204.2)	15.8 (1.4–115.5)	**<0.001**
Platelet count (exp9/L)	128.0 (36–487)	106.5 (14–344)	0.143
Serum sodium (mmol/L)	131.9 (119.1–147.5)	136.7 (116.7–143.7)	0.184
Ascites WBC (/μL)	1537.5 (437–17721)	146.5 (30–563)	**<0.001**
Ascites PMN (/μL)	1075.5 (281–14762)	15.5 (2–87)	**<0.001**
Ascites albumin (g/L)	6.9 (1–22)	5.8 (2–31)	0.802

SBP: Spontaneous bacterial peritonitis; ALD: Alcohol-related liver disease; MASH: Metabolic dysfunction-associated steatohepatitis; MELD: Model of end-stage liver disease; EF CLIF: European Foundation for the Study of Chronic Liver Failure; ACLF: Acute-on-chronic liver failure; PPI: Proton pump inhibitors; GFR: Glomerular filtration rate; INR: International normalized ratio; WBC: White blood count; CRP: C-reactive protein; PMN: Polymorphonuclear granulocytes. Bold value signifies P < 0.05.

### 16S rRNA Gene-Based PCR to Detect bactDNA in Ascites

In the SBP group, 24/42 (57.1%) of the ascites samples were positive for bactDNA, whereas microbial culture identified the causative pathogen in 17/42 (40.5%; *P* < 0.001) samples. All culture results were confirmed by PCR. In the non-SBP group, 5/56 (8.9%) of the ascites samples were positive for bactDNA despite negative bacterial cultivation (*P* = 0.003). Consequently, ascites bactDNA was detected more frequently in patients with SBP than in those without (*P* < 0.001).

We used PCR analysis of the bactDNA to identify the corresponding bacterial species as described earlier.^20^ Of 24 ascites samples from the SBP group, 13/24 (54.2%) samples contained predominantly Gram-positive bacteria, with *Streptococcus* as the most common genus, 8/24 (33.3%) samples contained predominantly Gram-negative bacteria, with *Escherichia* as the most common genus and 3/24 (12.5%) samples contained both, Gram-positive and Gram-negative bacteria. Of 5 ascites samples from the non-SBP group, 2/5 (40%) samples contained predominantly Gram-positive bacteria, with one case of *Staphylococcus* and *Lactococcus* each, 1/5 (20%) sample contained predominantly the Gram-negative bacterium *Escherichia*, and 2/5 (40%) samples contained both Gram-positive and Gram-negative bacteria (*P* = 0.284).

Levels of bactDNA in ascites were not different between the two groups (SBP 1.5 × 10^4^ copies/mL (3.6 × 10^2^–2.7 × 10^6^) vs non-SBP 3.7 × 10^3^ copies/mL (4.7 × 10^1^–6.7 × 10^3^); *P* = 0.157).

In analogy to ascites, significantly more blood samples were positive for bactDNA in patients with SBP (4/42; 9.5%), with a mean amount of 4.8 × 10^3^ copies/mL (8.2 × 10^2^–8.7 × 10^3^), compared to patients without SBP (0/56; 0%; *P* = 0.031) (Table 3). 2/4 (50%) samples of the SBP group contained Gram-positive bacteria of the genus *Streptococcus* and 2/4 (50%) samples contained Gram-negative bacteria of the genus *Escherichia* (Table 3).

**Table 3:** Comparison Between the Detection Rates of 16S rRNA Gene-Based PCR and Culture.

	SBP group (*n* = 42)	Non-SBP group (*n* = 56)	*P*-value
Blood PCR *Positive* *Quantity [copies/mL]*	4 (9.5%) 4.8 × 10^3^ (8.2 × 10^2^–8.7 × 10^3^)	0 (0%) n.a.	**0.031**
Ascites PCR *Positive* *Quantity [copies/mL]*	24 (57.1%) 1.5 × 10^4^ (3.6 × 10^2^–2.7 × 10^6^)	5 (8.9%) 3.7 × 10^3^ (4.7 × 10^1^–6.7 × 10^3^)	**<0.001** 0.157
Ascitic fluid culture *Positive*	17 (40.5%)	0 (0%)	**<0.001**
*P*_(Ascites PCR vs ascitic fluid culture)_	**<0.001**	**0.003**	

n.a., not applicable.

SBP: Spontaneous bacterial peritonitis; PCR: Polymerase chain reaction. Bold value signifies P < 0.05.

### Serum and Ascites Interleukin 6 Levels are Elevated in Patients With SBP

**Figure 1:**
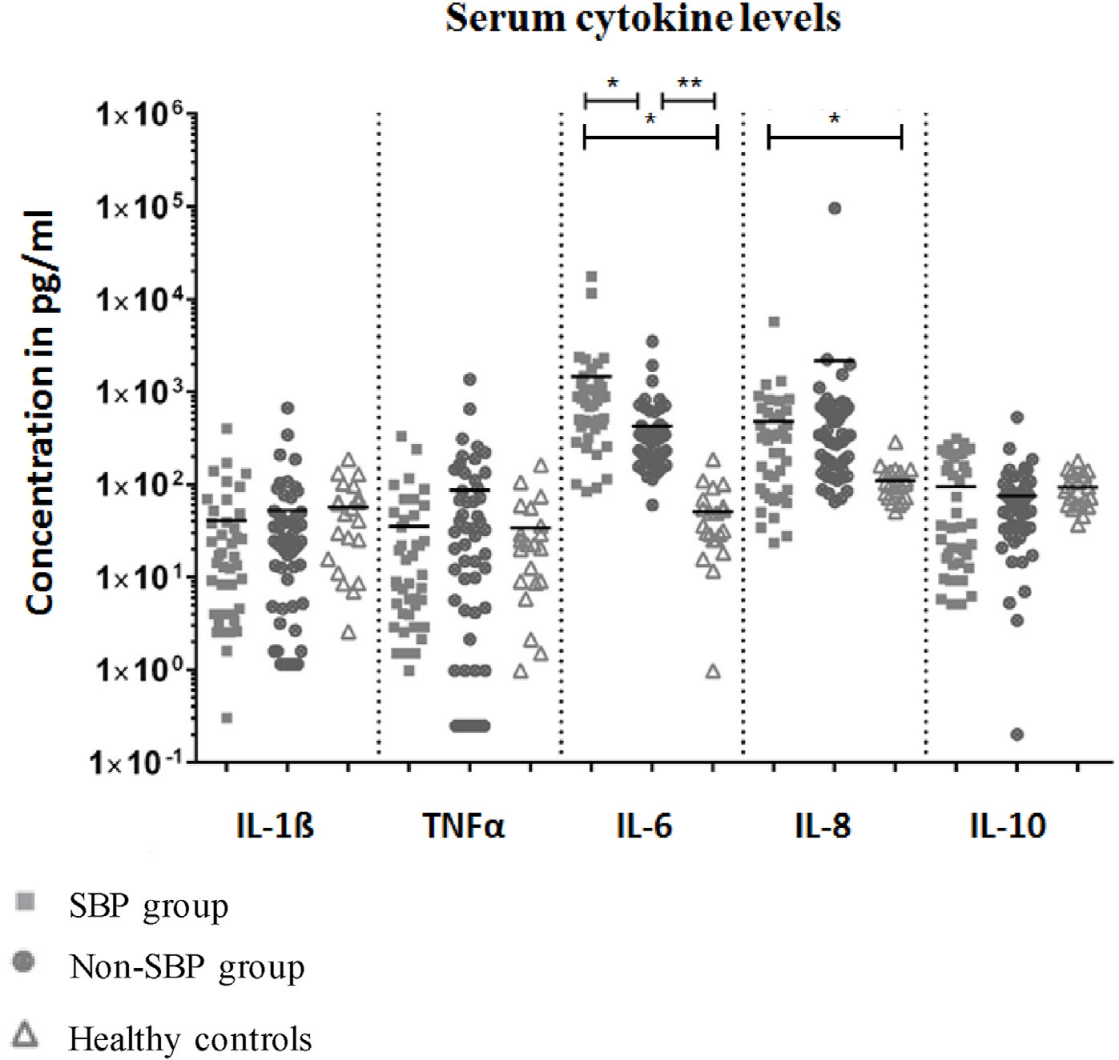
**Scatterplot of serum cytokine concentrations in patients with cirrhosis with or without SBP and healthy controls**. Levels of significance: ∗*P* < 0.05; ∗∗*P* < 0.001. SBP: Spontaneous bacterial peritonitis; IL: Interleukin; TNF: Tumor-necrosis-factor.

In order to explore potential differences of the immune response in patients with or without SBP, we measured serum cytokine levels (IL-1β, TNF-α, IL-6, IL-8 and IL-10). Serum IL-6 levels were increased in patients with SBP (733.4 pg/mL (83.4–17550.8)) compared to patients without SBP (306.1 pg/mL (59.8–3500.8); *P* = 0.036) and healthy controls (36.0 pg/mL (0.9–186.7); *P* = 0.005) (Figure 1). IL-8 levels were elevated in patients with SBP compared to healthy controls (305.1 pg/mL (23.6–5760.6) vs 98.5 pg/mL (51.1–287.2); *P* = 0.011) but not compared to patients without SBP (339.6 pg/mL (64.9–2230.4); *P* = 0.327). There were no differences in levels of IL-1β, TNF-α or IL-10 in serum (Figure 1).

**Figure 2:**
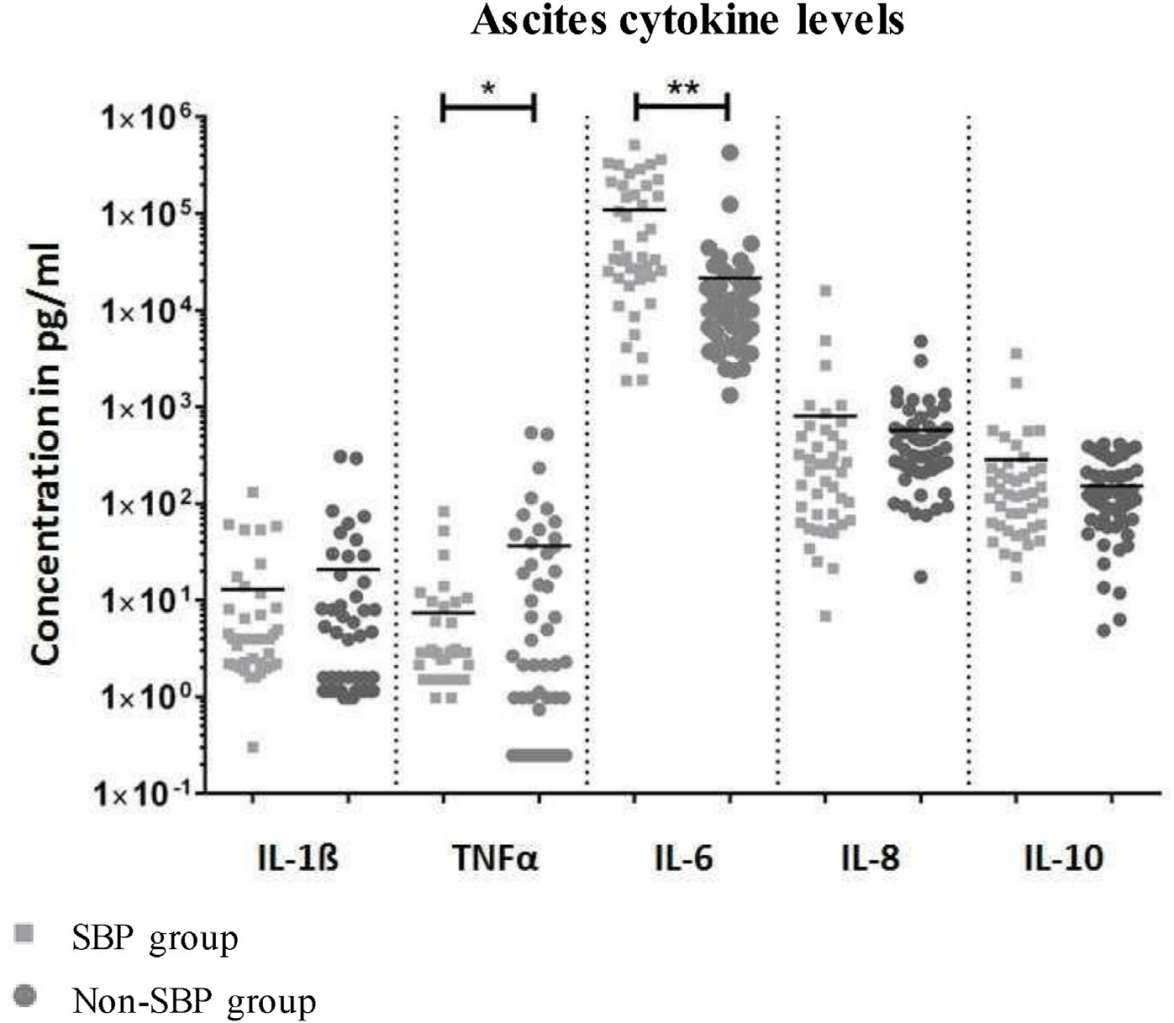
**Scatterplots for cytokine concentrations in ascites of patients with cirrhosis with or without SBP**. ∗*P* < 0.05; ∗∗*P* < 0.001. SBP: Spontaneous bacterial peritonitis; IL: Interleukin; TNF: Tumor-necrosis-factor.

Ascites IL-6 levels were higher in the SBP group (35293.2 pg/mL (1876.6–507929.7)) compared to the non-SBP group (9865.3 pg/mL (1319.0–427434.4); *P* < 0.001), whereas ascites TNF-α levels were lower in the SBP group compared to the non-SBP group (2.1 pg/mL (0.3–539.9) vs 2.9 pg/mL (0.9–82.9); *P* = 0.042) (Figure 2). BactDNA originating from Gram-negative bacteria did not lead to higher ascites or serum IL-6-levels. There were no significant differences in levels of IL-1β, IL-8 and IL-10 in ascites.

### BactDNA is Associated With Inflammatory Response

**Figure 3:**
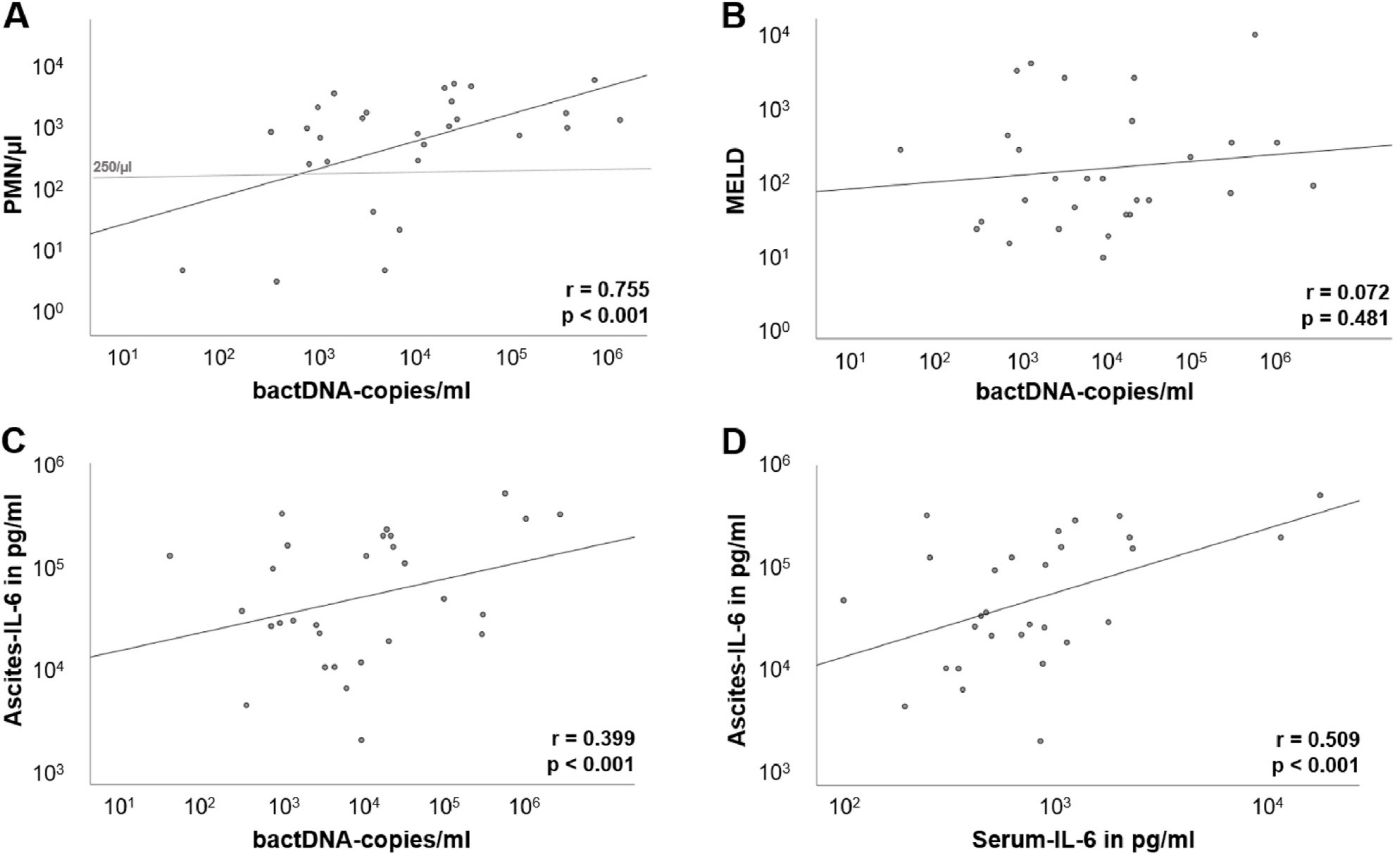
**Scatter plots showing various correlations**. (A–C) Correlations between the yield of ascites bactDNA and ascites PMN count (A), MELD (B) and ascites IL-6 levels (C). The horizontal line in diagram A marks the clinical cutoff of ascitic fluid PMN/μL in diagnosis of SBP (250/μL). (D) Correlation between the concentration of serum and ascites IL-6. bactDNA: bacterial DNA; PMN: polymorphonuclear granulocytes; MELD: Model of end-stage liver disease; IL: Interleukin; SBP: Spontaneous bacterial peritonitis.

Additional analyses showed a significant correlation between detection of bactDNA and inflammatory markers, with significantly higher levels of IL-6 in ascites in patients with detectable ascites bactDNA (35366.5 pg/mL (1900.9–507929.7) vs 10306.9 pg/mL (1319.0–427434.4); *P* = 0.010). Levels of other cytokines were not significantly different. Levels of bactDNA in ascites correlated strongly with ascites PMN count (*r* = 0.755; *P* < 0.001; Figure 3A), ascites IL-6 levels (*r* = 0.399; *P* < 0.001) (Figure 3C), PMN/mononuclear cell ratio (*r* = 0.690; *P* < 0.001) and ascites WBC (*r* = 0.557; *P* < 0.001). These correlations were enhanced considering patients with SBP only (Supplementary Figure 1). There was no correlation between ascites bactDNA levels and systemic inflammation markers (e.g. CRP, WBC). There was also no correlation between ascites bactDNA levels and MELD score (*r* = 0.072; *P* = 0.481) (Figure 3B). Ascites IL-6 levels correlated with serum IL-6 levels (*r* = 0.509; *P* < 0.001) (Figure 3D). Further correlations are listed in Supplementary Figure 1.

### Prophylactic Antibiotic Treatment Reduces Inflammation in Patients Without SBP

To assess a possible influence of antibiotic treatment on the results of this study, the group of patients with SBP was divided in groups with (*n* = 11) and without (*n* = 31) antibiotic treatment at time of paracentesis. There was no significant difference regarding baseline characteristics, cytokine levels, bactDNA quantities, differentiation of bacterial genera, the number of positive PCR results, and overall outcome. There was also no difference regarding the aforementioned parameters between patients with SBP and long-term prophylactic antibiotic treatment (*n* = 9) and patients with SBP without prophylactic antibiotic treatment (*n* = 33) (data not shown).

In the non-SBP group, patients with prophylactic antibiotic treatment (*n* = 6) had significant lower serum IL-6 levels (174.1 pg/mL (149.2–286.5) vs 322.1 pg/mL (59.8–3500.8); *P* = 0.002) than those without.

### Ascites Interleukin 6 is a Potential Tool in the Diagnosis of SBP

**Figure 4:**
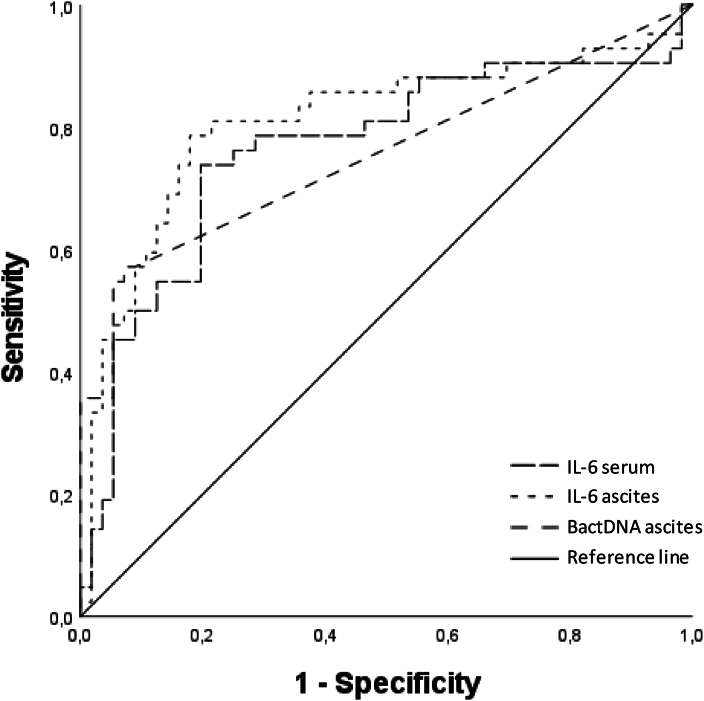
**ROC-analysis of IL-6 in serum and ascites as well as ascites bactDNA showing sensitivity and specificity in detecting cases of SBP**. Diagonal segments result from ties. ROC: receiver-operating-characteristic curve; bactDNA: bacterial DNA; IL: Interleukin; SBP: Spontaneous bacterial peritonitis.

ROC curve analysis was performed to measure the accuracy of serum and ascites cytokine levels to identify patients with SBP. Ascites IL-6 showed the largest AUC with a value of 0.810 (95% CI: 0.714–0.905), followed by serum IL-6 with an AUC of 0.764 (95% CI: 0.661–0.867). In further analysis, ascites bactDNA showed an AUC of 0.755 (95% CI: 0.651–0.858; Figure 4). Additional results are listed in Supplementary Table 3.

Focusing on those parameters, the optimal compromise for serum IL-6 in sensitivity and specificity was a cut-off value of 442.37 pg/mL, resulting in 73.8% sensitivity and 80.4% specificity. The optimal compromise for ascites IL-6 in sensitivity and specificity was a cut-off value of 2.0 × 10^5^ pg/mL, resulting in 78.6% sensitivity and 83.0% specificity.

Additionally, ROC curve analysis was applied to compare the value of aforementioned markers in predicting 30-day-mortality. Surprisingly, ascites IL-8 showed the best results with an AUC of 0.715 (95% CI: 0.567–0.862). Among the markers with the highest sensitivity/specificity in detection of SBP, the AUC of serum IL-6 was 0.696 (95% CI: 0.581–0.811), the AUC of ascites IL-6 was 0.635 (95% CI: 0.486–0.783) and the AUC of bactDNA was 0.579 (95% CI: 0.431–0.727). For the purpose of comparison, the calculated AUC of ascites fluid PMNs was 0.648 (95% CI: 0.523–0.773). The remaining results are listed in Supplementary Table 4.

In order to evaluate IL-6 as a potential marker to diagnose SBP, we performed two different survival analyses including all patients with cirrhosis despite their PMN count, divided by serum IL-6 and ascites IL-6 cut-offs as calculated before (serum IL-6: 442.37 pg/mL, ascites IL-6: 2.0 × 10^5^ pg/ml). 30 day-survival in patients with a serum IL-6 above 442.37 pg/mL was significantly lower than in patients with a serum IL-6 below 442.37 pg/mL (69 vs 91.1%; *P* = 0.01). There was no difference in survival using an ascites IL-6 of 2.0 × 10^5^ pg/mL as cut-off (data not shown).

### Effect of bactDNA Detection in Serum and Ascites on Survival

During the 360-day observation period, 40/98 (40.8%) patients died. The most common causes of death were liver failure (9/40; 22.5%) and sepsis (9/40; 22.5%), followed by bleeding (3/40; 7.5%) and non-classified causes (5/40 (12.5%)). In 14/48 (35%) cases, no data to determine the cause of death were available. 13/98 (13.3%) patients underwent orthotopic liver transplantation and 17/98 (17.3%) patients were lost to follow-up. The 30 day-mortality of patients with SBP was 26.2% (11/42) compared to 12.5% (7/56) for patients without SBP (*P* = 0.072). Univariate and multivariate cox regression adjusted for age, gender and MELD did not reveal PMNs as an independent risk factor for death within 30 days (Supplementary Table 2).

**Figure 5:**
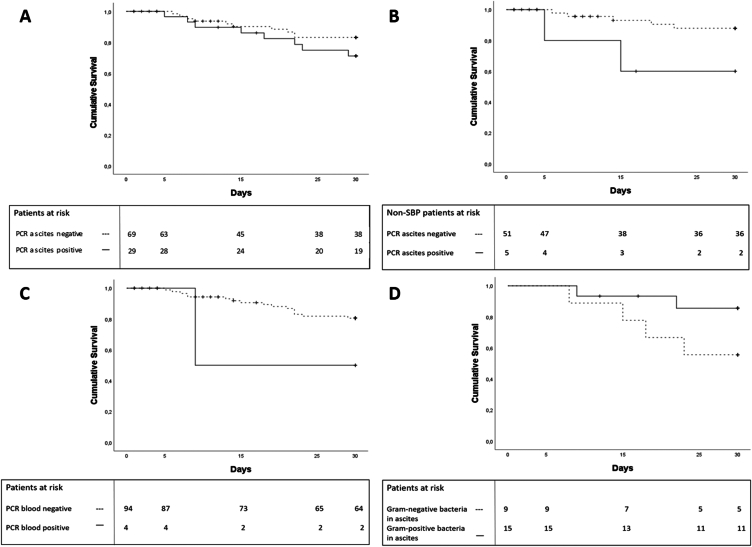
**Kaplan–Meier estimator regarding 30-day-survival**. (A) All patients with decompensated cirrhosis stratified by ascites bactDNA (*P* = 0.248). (B) All patients with ascites PMN count <250/μL stratified by ascites bactDNA (*P* = 0.042). (C) All patients with decompensated cirrhosis stratified by blood bactDNA (*P* = 0.048). (D) All patients with positive ascites bactDNA stratified by Gram-stain (*P* = 0.125). bactDNA: bacterial DNA; PMN: polymorphonuclear granulocytes.

Patients with bactDNA in ascites, as detected by PCR, had slightly, although not statistically significant, decreased survival (72.4%) compared to patients without bactDNA in ascites (85.5%; *P* = 0.248; Figure 5 A). Patients without SBP but with detectable ascites bactDNA had lower 30-day-survival (60%) than patients of the same group with undetectable bactDNA (90.2% survival; *P* = 0.042; Figure 5B). Taking all patients with decompensated cirrhosis into account, detection of bactDNA in bloodstream by PCR was associated with significant shorter 30-day survival (50% vs 82%; *P* = 0.048; Figure 5C). Patients with bactDNA from Gram-positive bacteria in ascites samples showed a tendency to higher 30-day-survival-rates (86.7%) than patients with bactDNA from Gram-negative bacteria in ascites samples (55.5%; *P* = 0.125; Figure 5D).

### Increasing Ascites Interleukin 6 is Associated With a Higher 30-day-mortality in Patients With Decompensated Cirrhosis

Univariate Cox regression analysis showed that an increased ascites IL-6 concentration was associated with higher risk of death within 30 days (HR 1.084, 95% CI: 1.000–1.174; *P* = 0.049). Univariate Cox regression analysis of ascites PMN count (HR 1.295, 95% CI: 0.853–1.965; *P* = 0.225) and ascites bactDNA levels (HR 1.049, 95% CI: 0.503–2.188; *P* = 0.899) did not show any association.

Multivariate Cox regression analysis adjusted for age, gender and MELD showed that an elevated ascites IL-6 concentration was associated with a higher 30-day-mortality (HR 1.008, 95% CI: 1.003–1.013; *P* = 0.004) in all patients with decompensated cirrhosis, whereas ascites PMN count and ascites bactDNA level did not show any significant association. Further results are listed in Supplementary Table 2.

## DISCUSSION

SBP is a common complication in cirrhosis and contributes to high mortality, especially if diagnosis and subsequent antibiotic therapy are delayed. Sensitivity and specificity of current diagnostic criteria are limited and novel markers may improve prognostic accuracy.

Therefore, we applied a 16S rRNA gene-based PCR method to detect, differentiate and quantify bactDNA in serum and ascitic fluid samples of 98 patients with decompensated cirrhosis. We also assessed the inflammatory response by measuring levels of pro- and anti-inflammatory cytokines in serum and ascites. We aimed to determine to what extent bactDNA leads to a local and systemic inflammatory response and thereby impacts disease outcome.

Even sterile ascites of patients with decompensated cirrhosis often contains IL-6 due to local macrophage priming.^22^ This explains higher ascites IL-6 levels compared to serum IL-6 even in non-SBP patients (Figure 1, Figure 2). In our study, both the presence of bactDNA in ascites as well as the quantity of bactDNA were correlated with increased IL-6 levels in ascites. In all patients with liver cirrhosis, the quantity of bactDNA in ascites correlated strongly with ascites markers of inflammation such as PMN count, ascites WBC and ascites IL-6 levels. We speculate that bactDNA might cause local (peritoneal) inflammation. Similarly, Zhang *et al.* described cytokine release triggered local presence of any kind of DNA via activation of cyclic guanosine monophosphate-adenosine monophosphate synthase (cGAS).^23^

Furthermore, among all the cytokines tested, ascites IL-6 levels exhibited the closest association with the presence of bacterial DNA in ascites. Data from patients with bacteremia suggest that bactDNA from Gram-negative bacteria correlates with elevated inflammation markers.^24^ By contrast, in our study, bactDNA origin did not correlate with differences in IL-6 levels. Other than IL-6, levels of other cytokines did not correlate with bactDNA abundance in our study, although a correlation between bactDNA and IL-8 was expected since IL-8 is known to mobilize granulocytes.^25^ Alvarez-Silva *et al.* also showed increased levels of IL-6 but not IL-8 being associated with the detection of bactDNA in patients with decompensated cirrhosis.^26^ One possibility is that bactDNA concentrations remained below a threshold necessary for cytokine release in our samples. Alternatively, there might be a difference between the immune reaction due to bactDNA originating from intact bacteria and damage- or pathogen-associated molecular patterns (DAMPs or PAMPs).

It is highly probable that our previously established PCR technique, which involves measuring bactDNA from ascites cell pellet samples and utilizes glycerol for storage, ensures that intact bacterial cells serve as the source of the bactDNA. It appears evident from our study findings that there exists a robust correlation between the measured bactDNA and cytokine levels, suggesting that bacterial pathogens, as identified through bactDNA measurement, induce an inflammatory response. However, it is important to acknowledge that the potential influence of DAMPs and PAMPs, with free circulating bacterial DNA possibly acting as PAMPs, was not assessed in our study.

Systemic inflammatory response triggered by bacterial infection might be the cause of worse clinical condition in patients with SBP. Indeed, others have suggested that high levels of cytokines are associated with higher risk of organ failure and thus mortality.^27^ Other publications, for example Hübener *et al.*, define local or systemic infections such as SBP as one of the main triggers of ACLF.^28^ In our cohort, SBP led to a significantly higher systemic, but especially local, inflammation in terms of IL-6 levels in serum and ascites (Figure 1).

We performed ROC analysis to measure the diagnostic accuracy of serum and ascites cytokine levels for detecting SBP. IL-6 appeared to be most promising of the tested cytokines, with high sensitivity and specificity for both serum IL-6 and ascites IL-6. The differences in the serum and ascites IL-6 thresholds that achieved high sensitivity and specificity likely reflect different regulation of IL-6 levels in serum and ascites.^29^ We also note that serum IL-6 is not a specific marker for SBP and might be associated with many inflammatory triggers including cancer, injury and other types of infections.

Mayr *et al.*^30^ showed that ascites IL-6 levels were associated with poor prognosis in a rather small cohort of 64 ICU patients, including 19 SBP patients. Here, ascites IL-6 levels showed an AUC of 0.802 to predict 3-month-mortality and an AUC of 0.901 to diagnose SBP. The AUC calculated with our data is not as good as described by Mayr *et al.*, but our cohort was larger by 50% and focused on patients, who were not critically ill, which might influence the results. Although the AUC in our study is lower, taken together these studies further suggest that IL-6 is a promising surrogate parameter in diagnosis of SBP compared to the established PMN count. Still, further studies are needed to define specific cut-offs and to evaluate ascites IL-6 in non-SBP infections.

Although bacterial cultivation still marks the gold standard in microbiological diagnosis of SBP,^31^ its low sensitivity limits its clinical application. We found a much higher detection rate of bactDNA by 16S rRNA PCR in patients with SBP compared with traditional culturing, providing a potential approach for optimized SBP diagnostics. Indeed, in addition to confirming all cases of positive microbial culture, one or more pathogens could be identified by PCR in an additional 16.6% of culture-negative ascites samples. Furthermore, PCR technology detected bactDNA in the non-SBP group in 8.9% of cases, although in none of these cases the culture was positive. The clinical significance of this finding is reflected in the fact that detection of bactDNA was also associated with higher ascites IL-6 levels as well as worse survival in the non-SBP group. It remains to be seen whether such patients tend to develop SBP over time, as has been described in the so-called bacterascites, and whether preemptive antibiotic treatment could affect their outcome.^7^

Survival rates of patients without SBP but with detectable bactDNA in ascites mirrored results from Engelmann *et al.*,^2^ who showed significantly lower 30-day survival in patients with quantifiable bactDNA (>5000 bactDNA copies/ml) compared to patients with low or undetectable bactDNA levels. These results further emphasize that ultraclean PCR techniques provide additional information on the link between bactDNA and outcome in cirrhosis. However, Bruns *et al.*,^32^ who collected and analyzed ascites and serum from 218 patients prospectively, had obtained different results. In their study, the detection rate of bactDNA was much higher than in our cohort. Further, Bruns *et al.* showed a correlation between the detection of bactDNA and disease severity with respect to ACLF grade and no difference in survival in patients with PMN <250/μL and positive bactDNA, which was not the case in our study. Several differences in study design may explain these discrepancies. First of all, Bruns *et al.* included only patients with an assumed infection, such that the pre-test probability of detecting bactDNA is higher. Secondly, they used a different PCR-test with a different target and no quantification.

Apart from quantity, the source of bactDNA appeared to influence mortality as well. The majority of ascites samples of the SBP group contained bactDNA from Gram-positive bacteria, with *Streptococcus* as the most common genus. Similarly, previous studies described a predominance of Gram-positive sequence types in ascites PCR^20^ and reported an increasing number of nosocomial SBP cases associated with Gram-positive bacteria^33^^,^^34^ as a main cause of infection. Interestingly, Alvarez-Silva *et al.*^26^ describe mainly bactDNA from Gram-negative bacteria. In any case, the source of bactDNA had no influence on cytokine levels in either study. We believe that the predominance of Gram-positive bacteria observed in our study correlates with the global trend of increased Gram-positive infections in cirrhosis patients. Nevertheless, our study's design precluded the identification of definitive confounding factors for this observation. Further analyses and data collection are necessary for a detailed exploration of this phenomenon.

In comparison to these findings, the quantification of IL-6 in ascites appears to be a well-performing tool in diagnosing SBP with the benefit of IL-6 quantification assays already being readily available for clinical use. The correlation between IL-6 levels and bactDNA yield provides additional information regarding the severity of infection and possibly worse outcome, especially in patients below the PMN cut-off in SBP diagnosis.

Still, like mentioned before, other potential triggers of inflammation have to be taken into account when trying to use ascites IL-6 concentration to diagnose SBP. For example, ascites IL-6 levels above the aforementioned cut-off were not correlated with worse outcome including all patients with cirrhosis despite their PMN count. This might be influenced by antibiotic treatment of patients with an elevated PMN count and the small sample size. Furthermore, to date, there is a lack of data regarding ascites IL-6 levels in patients afflicted with infections other than SBP. This gap necessitates further evaluation, as it may unveil a more accurate cut-off for diagnosing SBP.

Our study has several limitations. First, our study was retrospective with a rather small number of included patients, which limits statistical power and might particularly impact survival analysis. We did not include patients consecutively, since inclusion was only possible if there were ascites and serum samples at the same time, which might be a selection bias. Additionally, the number of patients in this study was too small to have a valid conclusion and needs further backup from a larger cohort collected in a prospective design. Based on our findings regarding the sensitivity/specificity of ascites IL-6 levels in diagnosis of SBP and assuming a SBP prevalence of ∼10% in patients with decompensated cirrhosis, we calculated a necessary sample size of at least 647 patients.

It would be particularly interesting to include more patients with less than 250 PMN/μL in ascites, not diagnosed with SBP, and detectable bactDNA by PCR to compare markers of inflammation, clinical data, possible complications, and survival with other cohorts like patients not diagnosed with SBP and negative bactDNA to further evaluate the usefulness of PCR and cytokine quantification as possible diagnostic tools. Apart from that, 11/42 patients of the SBP group received antibiotics during time of paracentesis to treat prior infections other than SBP. This might have influenced results of microbiological culture and as well the quantitative and qualitative results of PCR.

Cytokine levels, especially of IL-6, can vary substantially.^35^ Accordingly, we detected broad variation in cytokine concentrations, particularly for IL-6 in serum and ascites. Cytokine concentrations ranged from 10^1^ to 10^5^ pg/mL, which might affect the analytical precision of our applied cytokine assay. Although we used serum samples collected in a strictly defined 24-h window before or after paracentesis for quantification of cytokine levels, even a difference of a few hours might have caused a considerable shift in measured concentration. An even narrower window of sample collection would have helped, but it would have even further reduced our sample size and thus meaningfulness. It must be emphasized that multiplex assays present a promising avenue for enhancing data output from limited sample sizes.

The high sensitivity of the multiplex assay employed in this study underscores the importance of meticulous pre-analytical procedures to mitigate the risk of result distortion due to contamination. Additionally, addressing the challenge of signal-to-noise ratio and signal loss demands careful reagent titration, particularly in samples with lower cytokine levels. Previous publications have outlined general guidelines for flow cytometry antibody titration, which may inform and guide these pre-analytical efforts..^36^^,^^37^

In summary, using a bead-based flow assay and a qualitative and quantitative PCR method, we detected bactDNA in a significant proportion of our cohort and revealed possible links between cirrhosis and inflammation in patients with decompensated liver cirrhosis as well as a connection between bactDNA yield in ascites and immune response. Quantification of the cytokine IL-6 in ascitic fluid promises to be a valuable tool for detection of SBP and should be further evaluated.

## Credit authorship contribution statement

**Conceptualization:** Arno Hagenunger, Sandra Krohn, Niklas Aehling, Cornelius Engelmann, Thomas Berg.

**Data curation:** Arno Hagenunger, Sandra Krohn, Katharina Zeller, Kathrin Jäger.

**Formal analysis:** Arno Hagenunger, Niklas Aehling.

**Funding acquisition:** Thomas Berg, Jan Hartmann, Sandra Krohn, Stephan Böhm.

**Investigation:** Sandra Krohn, Cornelius Engelmann, Thomas Berg.

**Methodology:** Arno Hagenunger, Sandra Krohn, Katharina Zeller, Kathrin Jäger.

**Resources:** Thomas Berg, Stephan Böhm, Adam Herber, Albrecht Hoffmeister, Thorsten Kaiser.

**Supervision:** Thomas Berg.

**Visualization:** Arno Hagenunger, Anne Olbrich, Niklas Aehling.

**Writing – original draft:** Arno Hagenunger, Niklas Aehling, Sandra Krohn.

**Writing – review & editing:** Arno Hagenunger, Niklas Aehling, Sandra Krohn, Cornelius Engelmann, Thomas Berg.

## Conflicts of interest

The authors have none to declare.

## Acknowledgements

We thank Manuela Ehbrecht and Philine Geiβler for excellent technical support in the laboratory. For numerous suggestions and valuable advice, we thank Dr. Janett Fischer, Dr. Maria Pfefferkorn, Dr. Anne Olbrich and Alexandra Wodner.

## Funding

None.

## References

[1] Runyon B.A. (2003). The evolution of ascitic fluid analysis in the diagnosis of spontaneous bacterial peritonitis. Am J Gastroenterol.

[2] Engelmann C., Krohn S., Prywerek D. (2016). Detection of molecular bacterascites in decompensated cirrhosis defines a risk with decreased survival. Eur J Gastroenterol Hepatol.

[3] Niu B., Kim B., Limketkai B.N. (2018). Mortality from spontaneous bacterial peritonitis among hospitalized patients in the USA. Dig Dis Sci.

[4] Marciano S., Díaz J.M., Dirchwolf M., Gadano A. (2019). Spontaneous bacterial peritonitis in patients with cirrhosis: incidence, outcomes, and treatment strategies. Hepat Med.

[5] Rimola A., García-Tsao G., Navasa M. (2000). Diagnosis, treatment and prophylaxis of spontaneous bacterial peritonitis: a consensus document. International Ascites Club. J Hepatol.

[6] Castellote J., Girbau A., Maisterra S., Charhi N., Ballester R., Xiol X. (2008). Spontaneous bacterial peritonitis and bacterascites prevalence in asymptomatic cirrhotic outpatients undergoing large-volume paracentesis. J Gastroenterol Hepatol.

[7] Chu C.M., Chang K.Y., Liaw Y.F. (1995). Prevalence and prognostic significance of bacterascites in cirrhosis with ascites. Dig Dis Sci.

[8] Pelletier G., Lesur G., Ink O. (1991). Asymptomatic bacterascites: is it spontaneous bacterial peritonitis?. Hepatology.

[9] Pinzello G., Simonetti R.G., Craxì A., Di Piazza S., Spanò C., Pagliaro L. (1983). Spontaneous bacterial peritonitis: a prospective investigation in predominantly nonalcoholic cirrhotic patients. Hepatology.

[10] Runyon B.A. (1990). Monomicrobial nonneutrocytic bacterascites: a variant of spontaneous bacterial peritonitis. Hepatology.

[11] Runyon B.A., Hoefs J.C., Canawati H.N. (1986). Polymicrobial bacterascites. A unique entity in the spectrum of infected ascitic fluid. Arch Intern Med.

[12] Oey R.C., van Buuren H.R., de Jong D.M., Erler N.S., de Man R.A. (2018). Bacterascites: a study of clinical features, microbiological findings, and clinical significance. Liver Int.

[13] Perez I.C., Bolte F.J., Bigelow W., Dickson Z., Shah N.L. (2021). Step by step: managing the complications of cirrhosis. Hepat Med.

[14] Sajjad M., Khan Z.A., Khan M.S. (2016). Ascitic fluid culture in cirrhotic patients with spontaneous bacterial peritonitis. J Coll Phys Surg Pak.

[15] Gerbes A.L., Labenz J., Appenrodt B. (2019). [Updated S2k-guideline “Complications of liver cirrhosis”. German Society of Gastroenterology (DGVS)]. Zeitschr Gastroenterol.

[16] Zapater P., Francés R., González-Navajas J.M. (2008). Serum and ascitic fluid bacterial DNA: a new independent prognostic factor in noninfected patients with cirrhosis. Hepatology.

[17] El-Naggar M.M., Khalil E.A., El-Daker M.A.M., Salama M.F. (2008). Bacterial DNA and its consequences in patients with cirrhosis and culture-negative, non-neutrocytic ascites. J Med Microbiol.

[18] Bruns T., Sachse S., Straube E. (2009). Identification of bacterial DNA in neutrocytic and non-neutrocytic cirrhotic ascites by means of a multiplex polymerase chain reaction. Liver Int.

[19] Rogers G.B., van der Gast C.J., Bruce K.D. (2013). Ascitic microbiota composition is correlated with clinical severity in cirrhosis with portal hypertension. PLoS One.

[20] Krohn S., Böhm S., Engelmann C. (2014). Application of qualitative and quantitative real-time PCR, direct sequencing, and terminal restriction fragment length polymorphism analysis for detection and identification of polymicrobial 16S rRNA genes in ascites. J Clin Microbiol.

[21] European Association for the Study of the Liver (2018). Electronic address EEE, European Association for the Study of the L. EASL Clinical Practice Guidelines for the management of patients with decompensated cirrhosis. J Hepatol.

[22] Ruiz-Alcaraz A.J., Martínez-Esparza M., Caño R. (2011). Peritoneal macrophage priming in cirrhosis is related to ERK phosphorylation and IL-6 secretion. Eur J Clin Invest.

[23] Zhang X., Bai X.C., Chen Z.J. (2020). Structures and mechanisms in the cGAS-STING innate immunity pathway. Immunity.

[24] Abe R., Oda S., Sadahiro T. (2010). Gram-negative bacteremia induces greater magnitude of inflammatory response than gram-positive bacteremia. Crit Care.

[25] Harada A., Sekido N., Akahoshi T., Wada T., Mukaida N., Matsushima K. (1994). Essential involvement of interleukin-8 (IL-8) in acute inflammation. J Leukoc Biol.

[26] Alvarez-Silva C., Schierwagen R., Pohlmann A. (2019). Compartmentalization of immune response and microbial translocation in decompensated cirrhosis. Front Immunol.

[27] Kellum J.A., Kong L., Fink M.P. (2007). Understanding the inflammatory cytokine response in pneumonia and sepsis: results of the genetic and inflammatory markers of sepsis (GenIMS) study. Arch Intern Med.

[28] Hübener P., Braun G., Fuhrmann V. (2018). [Acute-on-chronic liver failure: a diagnostic and therapeutic challenge for intensive care]. Med Klin Intens Notfallmed.

[29] Zimmermann H.W., Reuken P.A., Koch A. (2013). Soluble urokinase plasminogen activator receptor is compartmentally regulated in decompensated cirrhosis and indicates immune activation and short-term mortality. J Intern Med.

[30] Mayr U., Lukas M., Elnegouly M. (2020). Ascitic interleukin 6 is associated with poor outcome and spontaneous bacterial peritonitis: a validation in critically ill patients with decompensated cirrhosis. J Clin Med.

[31] Pericleous M., Sarnowski A., Moore A., Fijten R., Zaman M. (2016). The clinical management of abdominal ascites, spontaneous bacterial peritonitis and hepatorenal syndrome: a review of current guidelines and recommendations. Eur J Gastroenterol Hepatol.

[32] Bruns T., Reuken P.A., Stengel S. (2016). The prognostic significance of bacterial DNA in patients with decompensated cirrhosis and suspected infection. Liver Int.

[33] Facciorusso A., Antonino M., Orsitto E., Sacco R. (2019). Primary and secondary prophylaxis of spontaneous bacterial peritonitis: current state of the art. Expet Rev Gastroenterol Hepatol.

[34] Piroth L., Pechinot A., Minello A. (2009). Bacterial epidemiology and antimicrobial resistance in ascitic fluid: a 2-year retrospective study. Scand J Infect Dis.

[35] Yuan S.M. (2019). Profiles and predictive values of interleukin-6 in aortic dissection: a review. Braz J Cardiovasc Surg.

[36] Brummelman J., Haftmann C., Núñez N.G. (2019). Development, application and computational analysis of high-dimensional fluorescent antibody panels for single-cell flow cytometry. Nat Protoc.

[37] Cossarizza A., Chang H.D., Radbruch A. (2019). Guidelines for the use of flow cytometry and cell sorting in immunological studies (second edition). Eur J Immunol.

