# Supplementary material for: Use of Bacterial DNA Concentration in Ascites as a Marker for Spontaneous Bacterial Peritonitis
Source: J Clin Exp Hepatol. 2024 May 1;14(5):101434. doi: 10.1016/j.jceh.2024.101434 (PMC11217685; doi:10.1016/j.jceh.2024.101434)
Supplement: Multimedia component 1 [file mmc1.docx]

**Use of bacterial DNA concentration in ascites as a marker for spontaneous bacterial peritonitis**

**Supplementary material**

Arno Hagenunger, Niklas Friedemann Aehling, Sandra Krohn, Katharina Zeller, Kathrin Jäger, Adam Herber, Cornelius Engelmann, Thomas Berg

**Table of contents:**

*List of contributions* …………………………………………………………………1

*Supplementary Fig. 1.*  …………………………………………………………………2

*Supplementary Table 1* …………………………………………………………………3

*Supplementary Table 2* …………………………………………………………………4

*Supplementary Table 3* …………………………………………………………………5

*Supplementary Table 4* ………………………………………………………................5

*Supplementary Table 5* ………………………………………………………............6-7

**Data curation:** Arno Hagenunger**,** Sandra Krohn, Katharina Zeller, Kathrin Jäger

**Formal analysis:** Arno Hagenunger, Niklas Aehling

**Investigation:** Sandra Krohn, Cornelius Engelmann, Thomas Berg

**Methodology:** Arno Hagenunger**,** Sandra Krohn, Katharina Zeller, Kathrin Jäger

**Visualization:** Arno Hagenunger, Niklas Aehling

**Supplementary Fig. 1**. **Correlations between bactDNA, local and systemic cytokines shown as a heat map. (**A) all patients with liver cirrhosis. (B) only patients with SBP were considered (C) only patients without SBP were considered. * p < 0.05; ** p < 0.001

**Supplementary Table 1: Bacterial primers utilized in 16S rRNA PCR of this study.**

| **Primer name** | **Direction** | **Sequences** |
| --- | --- | --- |
| 331F | Forward | TCCTACGGGAGGCAGCAGT |
| 797R | Reverse | GGACTACCAGGGTATCTAATCCTGTT |

**Supplementary Table 2: Univariate and multivariate Cox regression analysing the risk of death within 30 days after sample collection adjusted for age, gender and MELD.**

|  | **Hazard Ratio (95%CI)**  **Univariate Analysis** | **Hazard Ratio (95%CI)**  **Multivariate Analysis** | **Hazard Ratio (95%CI)**  **Univariate Analysis** | **Hazard Ratio (95%CI)**  **Multivariate Analysis** | **Hazard Ratio (95%CI)**  **Univariate Analysis** | **Hazard Ratio (95%CI)**  **Multivariate Analysis** |  |
| --- | --- | --- | --- | --- | --- | --- | --- |
|  | **All patients (n=98)** | | **SBP group (n=42)** | | **Non-SBP group (n=56)** | |  |
| **Risk of death within 30 days after sample collection** | | | | | | | |
| **PMN (a)** | 0.999 (0.996-1.002)  p=0.675 | 0.996 (0.991-1.000)  p=0.060 | 0.999 (0.996-1.003)  p=0.597 | 0.998 (0.993-1.002)  p=0.301 | 1.295 (0.853-1.965)  p=0.225 | 1.822 (0.919-3.615)  p=0.086 |  |
| **bactDNA  quantity (a)** | 1.000 (0.998-1.002)  p=0.938 | 1.000 (0.997-1.002)  p=0.820 | 1.000 (0.998-1.002)  p=0.780 | 1.000 (0.997-1.002)  p=0.834 | 1.049 (0.503-2.188)  p=0.899 | 2.557 (0.899-7.276)  p=0.078 |  |
| **IL-6 (a)** | 1.004 (1.000-1.007)  p=0.056 | **1.008 (1.003-1.013)**  **p=0.004** | 1.003 (0.998-1.007)  p=0.233 | 1.005 (0.999-1.010)  p=0.087 | **1.084 (1.000-1.174)**  **p=0.049** | 1.111 (0.984-1.253)  p=0.088 |  |

**Supplementary Table 3: ROC analysis of different cytokines regarding the diagnosis of SBP.**

| Cytokine | AUC | 95% CI |
| --- | --- | --- |
| IL-1B ascites | 0.627 | 0.512 – 0.742 |
| TNF-alpha ascites | 0.580 | 0.462 – 0.699 |
| IL-10 ascites | 0.525 | 0.407 – 0.643 |
| IL-10 serum | 0.473 | 0.342 – 0.604 |
| TNF-alpha serum | 0.467 | 0.352 – 0.583 |
| IL-1B serum | 0.449 | 0.332 – 0.565 |
| IL-8 serum | 0.407 | 0.290 – 0.524 |
| IL-8 ascites | 0.337 | 0.222 – 0.451 |

**Supplementary Table 4: ROC analysis of different cytokines regarding the prediction of 30-day-mortality.**

| Cytokine | AUC | 95% CI |
| --- | --- | --- |
| IL-1B ascites | 0.681 | 0.542 – 0.819 |
| IL-8 serum | 0.663 | 0.507 – 0.820 |
| IL-1B serum | 0.642 | 0.522 – 0.762 |
| IL-10 serum | 0.623 | 0.477 – 0.768 |
| TNF-alpha ascites | 0.560 | 0.437 – 0.683 |
| TNF-alpha serum | 0.547 | 0.408 – 0.687 |
| IL-10 ascites | 0.533 | 0.388 – 0.679 |

#### Supplementary Table 5. STARD* 2015 List of Essential Items for Reporting Diagnostic Accuracy Studies.

| Section and topic | Number | Item | Page(s) |
| --- | --- | --- | --- |
| **Title or abstract** |  |  |  |
|  | 1 | Identification as a study of diagnostic accuracy using at least one measure of accuracy (such as sensitivity, specificity, predictive values, or AUC) | 3-4 |
| **Abstract** |  |  |  |
|  | 2 | Structured summary of study design, methods, results, and conclusions | 3-4 |
| **Introduction** |  |  |  |
|  | 3 | Scientific and clinical background, including the intended use and clinical role of the index test | 4-5 |
|  | 4 | Study objectives and hypotheses | 5 |
| **Methods** |  |  |  |
| Study design | 5 | Whether data collection was planned before the index test and reference standard were performed (prospective study) or after (retrospective study) | 5 |
|  | 6 | Eligibility criteria | 5-6 |
| Participants | 7 | On what basis potentially eligible participants were identified (such as symptoms, results from previous tests, inclusion in registry) | 5-6 |
|  | 8 | Where and when potentially eligible participants were identified (setting, location, and dates) | 5-6 |
|  | 9 | Whether participants formed a consecutive, random, or convenience series | 5-6 |
| Test methods | 10a | Index test, in sufficient detail to allow replication |  |
|  | 10b | Reference standard, in sufficient detail to allow replication | n.s. |
|  | 11 | Rationale for choosing the reference standard (if alternatives exist) | 4 |
|  | 12a | Definition of and rationale for test positivity cut-offs or result categories of the index test, distinguishing pre-specified from exploratory | 12-13 |
|  | 12b | Definition of and rationale for test positivity cut-offs or result categories of the reference standard, distinguishing pre-specified from exploratory | 4 |
|  | 13a | Whether clinical information and reference standard results were available to the performers or readers of the index test | n.s. |
|  | 13b | Whether clinical information and index test results were available to the assessors of the reference standard | n.s. |
|  | 14 | Methods for estimating or comparing measures of diagnostic accuracy | 8, 12-13 |
|  | 15 | How indeterminate index test or reference standard results were handled | n.s. |
|  | 16 | How missing data on the index test and reference standard were handled | n.a. |
|  | 17 | Any analyses of variability in diagnostic accuracy, distinguishing pre-specified from exploratory | 12-13 |
|  | 18 | Intended sample size and how it was determined | 6 |
| **Results** |  |  |  |
| Participants | 19 | Flow of participants, using a diagram | n.s. |
|  | 20 | Baseline demographic and clinical characteristics of participants | 22-24 |
|  | 21a | Distribution of severity of disease in those with the target condition | 23 |
|  | 21b | Distribution of alternative diagnoses in those without the target condition | 23 |
|  | 22 | Time interval and any clinical interventions between index test and reference standard | 6 |
| Test results | 23 | Cross tabulation of the index test results (or their distribution) by the results of the reference standard | Supp. mat. page 2 |
|  | 24 | Estimates of diagnostic accuracy and their precision (such as 95% confidence intervals) | 12-13 |
|  | 25 | Any adverse events from performing the index test or the reference standard | n.a. |
| **Discussion** |  |  |  |
|  | 26 | Study limitations, including sources of potential bias, statistical uncertainty, and generalisability | 19-20 |
|  | 27 | Implications for practice, including the intended use and clinical role of the index test | 18, 20 |
| **Other Information** |  |  |  |
|  | 28 | Registration number and name of registry | n.a. |
|  | 29 | Where the full study protocol can be accessed | n.a. |
|  | 30 | Sources of funding and other support; role of funders | 2 |

*Standards for Reporting Diagnostic accuracy studies; Bossuyt PM et al. Clinical Chemistry. 2015. pii: clinchem.2015.246280. PMID: 26510957

n.s., not stated

n.a., not applicable
